# CT imaging features of lung ground-glass nodule patients with upgraded intraoperative frozen pathology

**DOI:** 10.1007/s12672-024-00872-x

**Published:** 2024-02-04

**Authors:** Hongya Wang, Aiping Chen, Kun Wang, He Yang, Wei Wen, Qianrui Ren, Liang Chen, Xinfeng Xu, Quan Zhu

**Affiliations:** 1 Department of Thoracic Surgery, The First Affiliated Hospital of Nanjing Medical University, 300 Guangzhou Road, Nanjing, 210029 China; 2 Department of Radiology, The First Affiliated Hospital of Nanjing Medical University, 300 Guangzhou Road, Nanjing, 210029 China; 3 Department of Thoracic Surgery, The Second Affiliated Hospital of Soochow University, Suzhou, 215004 China

**Keywords:** Frozen section pathology, Non-small cell lung cancer, Ground-glass nodule, Computed tomography

## Abstract

**Purpose:**

Intraoperative frozen section pathology (FS) is widely used to guide surgical strategies while the accuracy is relatively low. Underestimating the pathological condition may result in inadequate surgical margins. This study aims to identify CT imaging features related to upgraded FS and develop a predictive model.

**Methods:**

Collected data from 860 patients who underwent lung surgery from January to December 2019. We analyzed the consistency rate of FS and categorized the patients into three groups: Group 1 (n = 360) had both FS and Formalin-fixed Paraffin-embedded section (FP) as non-invasive adenocarcinoma (IAC); Group 2 (n = 128) had FS as non-IAC but FP as IAC; Group 3 (n = 372) had both FS and FP as IAC. Clinical baseline characteristics were compared and propensity score adjustment was used to mitigate the effects of these characteristics. Univariate analyses identified imaging features with inter-group differences. A multivariate analysis was conducted to screen independent risk factors for FS upgrade, after which a logistic regression prediction model was established and a receiver operating characteristic (ROC) curve was plotted.

**Results:**

The consistency rate of FS with FP was 84.19%. 26.67% of the patients with non-IAC FS diagnosis were upgraded to IAC. The predictive model’s Area Under Curve (AUC) is 0.785. Consolidation tumor ratio (CTR) ≤ 0.5 and smaller nodule diameter are associated with the underestimation of IAC in FS.

**Conclusion:**

CT imaging has the capacity to effectively detect patients at risk of upstaging during FS.

## Introduction

According to the American Cancer Society, lung cancer remains the second most prevalent and the leading cause of death from malignant tumors [1]. With the routine use of low-dose CT scans for physical examinations, an increasing number of early-stage lung cancers are detected [2]. Lobectomy is the standard procedure for treating lung cancer. The conflict between radical tumor removal and the patient’s desire to retain healthy lung tissue has led to studies such as JCOG0201, JCOG0802, and JCOG0804 on the prognosis of sublobar resection versus lobectomy [3–5].

The 5-year postoperative survival rate for adenocarcinoma in situ (AIS) and minimally invasive adenocarcinoma (MIA) patients reaches or approaches 100%, while the prognosis for IAC is relatively poorer [6]. According to the classification criteria of IASLC/ATS/ERS from 2011, atypical adenomatous hyperplasia (AAH), distinct from AIS, MIA, and IAC, corresponds to squamous dysplasia and typically measures less than 5 mm [7]. Some studies suggest that the treatment time window for lung cancer includes the AIS and MIA stages in pathology [8]. Surgical intervention is not recommended for AAH. Therefore, this study excluded nodules with pathological results indicating AAH.

Sublobar resection is suitable for AIS/MIA patients [9], whereas the surgical approach for IAC patients is more complicated, possibly related to factors like nodule size [10, 11]. This requires surgeons to select appropriate surgical methods based on potential pulmonary nodule pathology. Studies have confirmed that preparing rapid intraoperative frozen sections and deciding whether to expand the surgical plan based on intraoperative pathology results can effectively guide surgical procedures and have been widely adopted [12, 13]. FS can effectively identify early lesions [14], opting for a more conservative surgical approach to protect the patient’s healthy lung tissue [15]. According to the JCOG series studies results, nodules with a diameter greater than 3 cm should undergo pulmonary lobectomy. In comparison nodules with a diameter of 3 cm or less should have their surgical approaches determined with CTR [16]. The choice of surgical approach for such nodules can be challenging, and the accuracy of FS is particularly crucial in these cases.

However, FS has some errors due to the histological heterogeneity of lung adenocarcinoma, errors in sampling and interpretation, and suboptimal sample quality, among others. Due to variations in the expertise level among pathologists in different medical institutions, the accuracy of FS varies significantly [14, 17–19]. Much research currently predicts nodule infiltration levels through CT imaging characteristics, revealing that predicting IAC patients using CT features like the nodule diameter, CTR, or radiomics has a high accuracy rate [20, 21]. This research aims to study the CT imaging characteristics of FS upgrade patients, identify lung nodule patients prone to FS upgrades.

## Methods

This research project was approved by the First Affiliated Hospital of Nanjing Medical University. Individual patient informed consent was waived due to the anonymous data management and the retrospective nature of this study.

### Patients

This research involves a retrospective analysis. The study retrospectively analyzed patients who underwent lung surgery at the First Affiliated Hospital of Nanjing Medical University from January to December 2019. Inclusion and exclusion criteria were set as follows:

Inclusion Criteria:

1. Postoperative pathology suggested adenocarcinoma;
2. No neoadjuvant treatment before surgery;
3. Only a single lung nodule was operated on, or for multiple nodules, only the one with the highest degree of infiltration was studied.

**Fig. 1:**
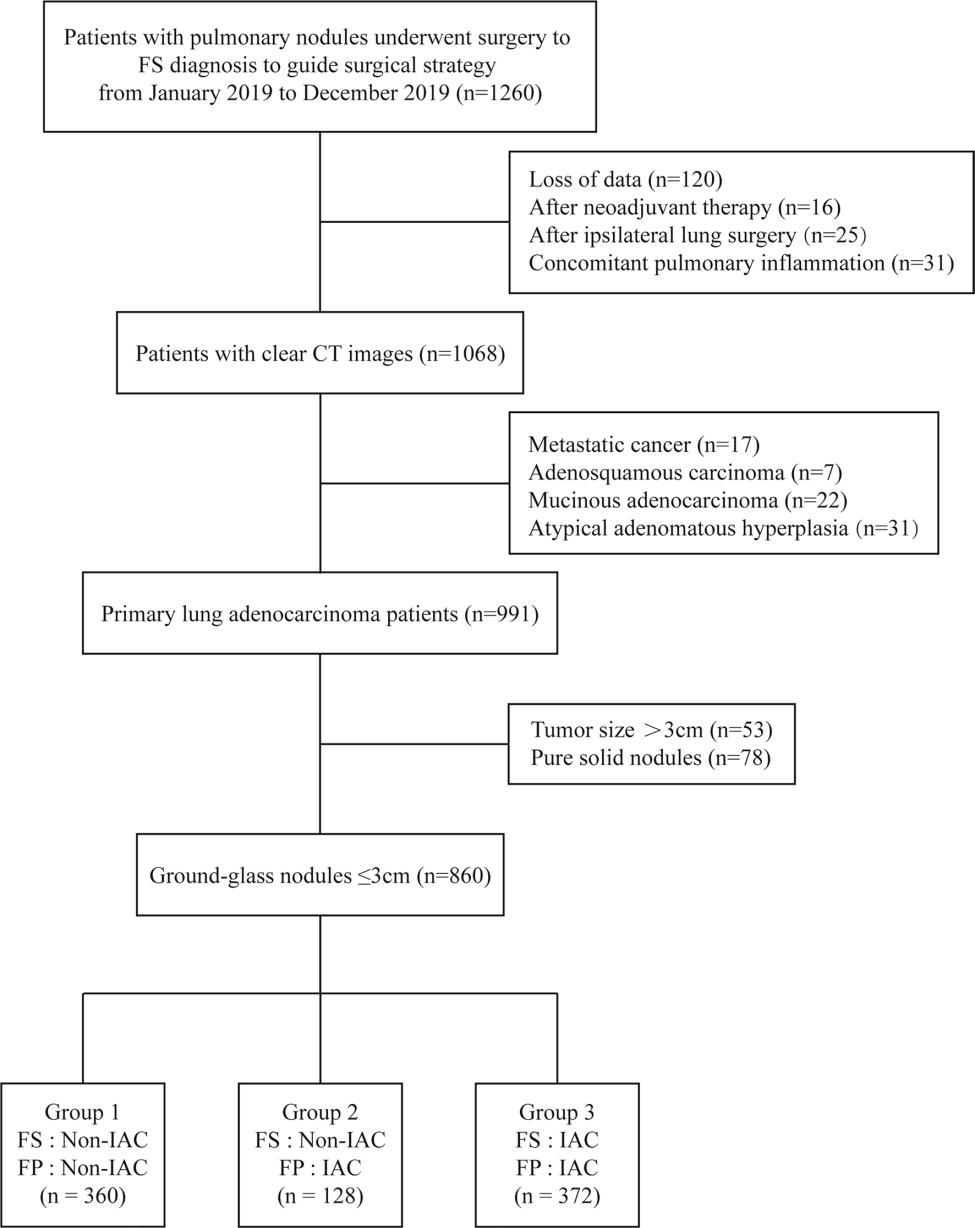
Patients’ recruitment process

Exclusion Criteria:

1. Incomplete medical records or imaging data.
2. Patients with other lung diseases affecting the measurement accuracy such as pneumonia, interstitial lung disease, tuberculosis, etc.;
3. Previous lung surgery on the same side;
4. Metastatic tumors from other organs in the lung;
5. Pathology results as AAH;
6. Purely solid nodules;
7. Nodule diameter > 3 cm;

1260 lung adenocarcinoma patients were included, with 120 excluded due to lost medical or imaging records, 16 after neoadjuvant therapy, 25 with surgery on the same side of the lung, 31 with lung inflammation, 17 with metastatic cancer, 7 with adenosquamous carcinoma, 22 with mucinous adenocarcinoma, 31 with AAH pathology results, 78 with purely solid nodules, and 53 with nodules larger than 3 cm in diameter. The remaining 860 patients were included in the study. For the purposes of this research, patients were divided into the following three groups: Group 1 (FS as non-IAC) 360 cases, Group 2 (FS as non-IAC) 128 cases, Group 3 (both FS and FP as IAC) 372 cases. (Fig. 1).

### Data collection procedures

#### Evaluation of radiologic findings

The imaging software was set to a lung window width of 1,600 Hu and window level of -600 Hu. Two radiologists specializing in thoracic imaging and one thoracic surgeon jointly reviewed the images. Before reviewing, the data was anonymized; the three doctors were unaware of the patient’s basic information, surgical plan, and pathological results. The diameter of the nodule and the solid component were measured in the cross-sectional view, and the average was taken from the measurements made by the three doctors. The CTR was calculated as the diameter of the solid component divided by the diameter of the nodule, following the measurement standards recommended by the Fleischner Society [22].

#### Evaluation of intraoperative FS results and FP findings

The diagnosis of the pathological results was based on the classification criteria of IASLC/ATS/ERS from 2011[7].Clinical information and imaging data are available when it’s necessary.

### Statistical analysis

All statistical analyses and prediction model were completed using the R language. Firstly, univariate analysis was conducted on the patients’ clinical baseline characteristics. When there was significant difference, a 1:2 propensity score matching was used to balance the baseline characteristics. Univariate analysis was used to filter radiological features with group differences. Quantitative data were tested using t-tests, while quantitative data were subjected to a χ^2^ test. Features with a P value less than 0.05 were included in the multivariate analysis to identify independent risk factors affecting outcomes. Specifically, when comparing Group 1 with Group 2 in order to build a prediction model, LASSO regression was employed to select independent risk factors and to simplify the model. Logistic regression was then used to build the prediction model, and ROC curves and nomograms were plotted. When comparing Group 2 with Group 3, to retain all potential independent risk factors, logistic regression was applied to select independent risk factors and calculated the AUC and cut-off values for continuous variables. A P value less than 0.05 was considered statistically significant.

## Results

### Consistency between FS and FP

The study included a total of 860 patients. Among these, 614 patients had consistent pathology results between FS and FP, yielding an overall accuracy rate of 71.40%. When we grouped AAH, AIS, and MIA as the non-IAC category, the overall FS accuracy rate reached 84.19%. For IAC patients, the accuracy rate was 74.40%. For those diagnosed as non-IAC, the FS upgrade rate was 26.67%. (Table 1).

**Table 1:** Conformance rate between FS with FP

FS/FP		AAH	AIS	MIA	IAC	Accuracy (%)	Non-IAC	IAC	Accuracy (%)
Non-IAC	AIS	7	69	14	0	76.67	352	8	97.22
	MIA	6	83	173	8	64.07			
IAC		0	24	104	372	74.40	128	372	74.40

### Correlation between FS upgrades and CT imaging

#### Group 1 vs group 2

**Fig. 2:**
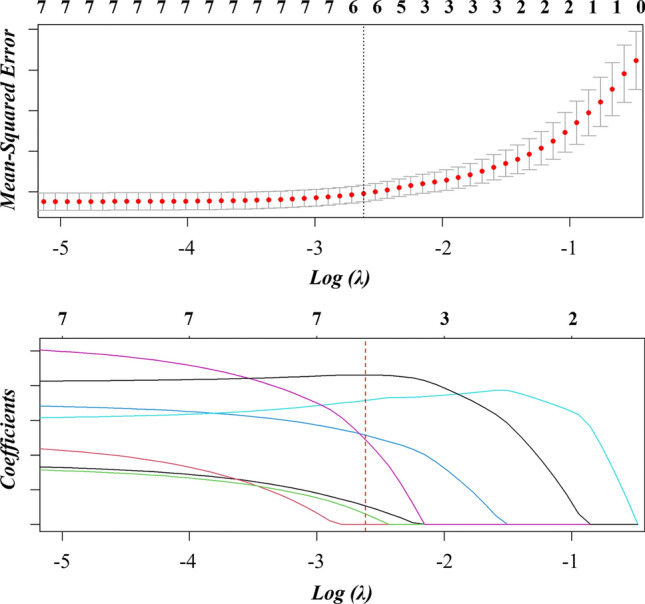
Lasso regression selecting independent risk factors, with Log(λ) being 2.619

**Fig. 3:**
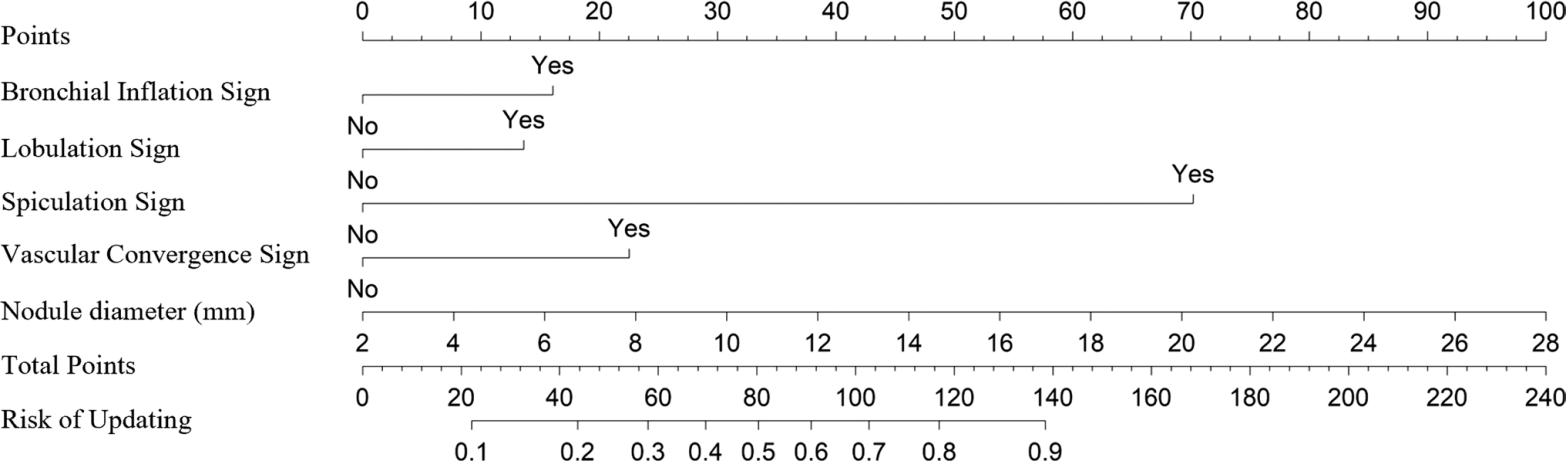
Nomogram for predicting frozen section pathology upgrade

Initially, patients were matched 1:2 based on gender, age, body mass index (BMI), and smoking history using propensity scores to reduce confounding factors. After matching, Group 1 included 211 cases, and Group 2 included 121 cases. There was no significant difference in the four features after matching. (Table 2) After matching, the two groups were subjected to univariate analysis, comparing features such as bronchial inflation signs, bubble signs, vascular convergence signs, lobulation signs, spiculation signs, pleural traction signs, CTR > 0.5, and the diameter of nodules. The results showed significant statistical differences between the two groups in the bronchus inflation sign (P < 0.001), vascular convergence sign (P < 0.001), lobulation sign (P < 0.001), spiculation sign (P < 0.001), pleural traction sign (P = 0.001), CTR > 0.5 (P = 0.001), and the diameter of nodule (P < 0.001). These features were incorporated into the LASSO regression. (Fig. 2) The coefficients for the pleural traction sign and CTR > 0.5 were 0. (Table 3) After filtering, the remaining five features were used to construct a predictive model using binary Logistic regression. An ROC curve was plotted with an AUC of 0.785 and an accuracy of 0.726. Based on this predictive model, a nomogram was drawn. (Fig. 3).

**Table 2:** Group 1 and Group 2 before and after matching

		Before			After		
		Group 1	Group 2	P	Group 1	Group 2	P
Gender	M	100	44	0.160	66	41	0.625
	F	260	84		145	80	
Age	< 60	275	64	< 0.001*	126	64	0.227
	≥ 60	85	64		85	57	
BMI	< 24	249	76	0.044*	142	74	0.259
	≤ 24	111	52		69	47	
Smoking	N	335	113	0.091*	195	109	0.461
	Y	25	15		16	12	

Marked with * indicate statistical significance

**Table 3:** Univariate and multivariate analysis between group 1 and group 2

		Group 1	Group 2	P	LASSO Coefficient
Bronchial inflation sign	N	155	55	< 0.001*	0.041
	Y	56	66		
Bubble sign	N	126	61	0.100	/
	Y	85	60		
Vascular convergence sign	N	128	31	< 0.001*	0.105
	Y	83	90		
Lobulation sign	N	93	23	< 0.001*	0.002
	Y	118	98		
Spiculation sign	N	210	105	< 0.001*	0.139
	Y	1	16		
Pleural traction sign	N	161	72	0.001*	0
	Y	50	49		
CTR	≤ 0.5	196	98	0.001*	0
	> 0.5	15	23		
Diameter of nodule (mm)		9.37(± 3.58)	12.78(± 4.71)	< 0.001*	0.078

Marked with * indicate statistical significance

#### Group 2 vs group 3

When comparing the features between Group 2 and Group 3, the results revealed statistically significant differences in the signs of bronchial inflation sign (P < 0.001), vascular convergence sign (P = 0.010), lobulation sign (P < 0.001), pleural traction sign (P = 0.003), CTR > 0.5 (P < 0.001), and the diameter of nodule (P < 0.001) between the two groups. These features were included in the binary logistic regression, and ultimately, CTR > 0.5 and the diameter of nodule were identified as independent risk factors for underestimating IAC patients. CTR ≤ 0.5 and smaller nodule diameter are associated with the underestimation of IAC in FS. The AUC for the diameter of nodule was 0.681, with a cut-off value of 12.55 mm. (Table 4).

**Table 4:** Univariate and multivariate analysis between group 2 and group 3

		Group 2	Group 3	P	Logistic	
					P	OR
Gender	M	44 (34.4%)	116 (31.2%)	0.504	/	/
	F	84 (65.6%)	256 (68.8%)			
Age	< 60	64 (50%)	185 (49.7%)	0.958	/	/
	≥ 60	64 (50%)	187 (50.3%)			
BMI	< 24	76 (59.4%)	208 (55.9%)	0.495	/	/
	≤ 24	52 (40.6%)	164 (44.1%)			
Smoking	N	113 (88.3%)	329 (88.4%)	0.961	/	/
	Y	15 (11.7%)	43 (11.6%)			
Bronchial inflation sign	N	59 (46.1%)	101 (27.2%)	< 0.001*	0.180	/
	Y	69 (53.9%)	271 (72.8%)			
Bubble sign	N	62 (48.4%)	200 (53.8%)	0.298	/	/
	Y	66 (51.6%)	172 (46.2%)			
Vascular convergence sign	N	33 (25.8%)	58 (15.6%)	0.010*	0.852	/
	Y	95 (74.2%)	314 (84.4%)			
Lobulation sign	N	24 (18.8%)	27 (7.3%)	< 0.001*	0.057	/
	Y	104 (81.3%)	345 (92.7%)			
Spiculation sign	N	112 (87.5%)	315 (84.7%)	0.649	/	/
	Y	16 (12.5%)	56 (15.1%)			
Pleural traction sign	N	77 (60.2%)	168 (45.2%)	0.003*	0.703	/
	Y	51 (39.8%)	204 (54.8%)			
CTR	≤ 0.5	103 (80.5%)	231 (62.1%)	< 0.001*	0.007*	0.500
	> 0.5	25 (19.5%)	141 (37.9%)			
Diameter of nodule(mm)		12.70 (± 4.69)	16.19 (± 5.79)	< 0.001*	< 0.001*	0.888

Marked with * indicate statistical significance

## Discussion

According to the data from our center, the accuracy of FS for lung adenocarcinoma is 71.40%. When we grouped AAH, AIS, and MIA as the non-IAC category, the overall FS accuracy rate reached 84.19%. In the meta-analysis conducted by Li et al., the concordance rate between FS and FP reached 95% [14]. Similarly, in the study by Zhang et al., this proportion was 93.7% [23], significantly higher than our findings. Apart from differences in practical factors such as inter-center pathological diagnostic capabilities, we consider the possible reason for this variance in results. We focused our study on nodules posing diagnostic and surgical challenges. Therefore, we excluded a considerable number of nodules with a diameter > 3 cm or pure solid nodules during subject selection. Due to their higher malignancy, these nodules are more likely to be diagnosed as IAC during FS, thus enhancing the overall accuracy. This perspective is supported by a study focusing on FS for AIS/MIA, where the concordance rate between FS and FP was only 63.24% [18],seemingly confirming our viewpoint. About 26.67% of patients diagnosed with AAH/AIS/MIA by FS are upgraded to IAC upon FP diagnosis. Among IAC patients, approximately 25.6% are underestimated during FS diagnosis. This group might have been chosen for sub-lobar resection based on intraoperative FS, resulting in inadequate surgical margins. The necessity for a second surgery remains controversial [23, 24]. In the study by Zhang et al. [23], patients who were upgraded from AAH/AIS/MIA to IAC had 5-year survival and relapse-free rates of 100%, regardless of whether they underwent a second surgery. In the study by Su et al. [25] two patients who underwent sub-lobar resection and were upgraded post-operatively experienced local recurrence. They believed that a second surgery was necessary for patients with FS upgrade.

The optimal solution to this problem is to improve the accuracy of FS. Some studies indicate that sampling errors during slide preparation are the main factor affecting FS accuracy [26]. However, meticulously studying each pathological specimen would consume a lot of pathologist’s time and energy and would not meet clinical needs. Hence, it’s vital to focus on patients where FS is prone to misdiagnosis.

**Fig. 4:**
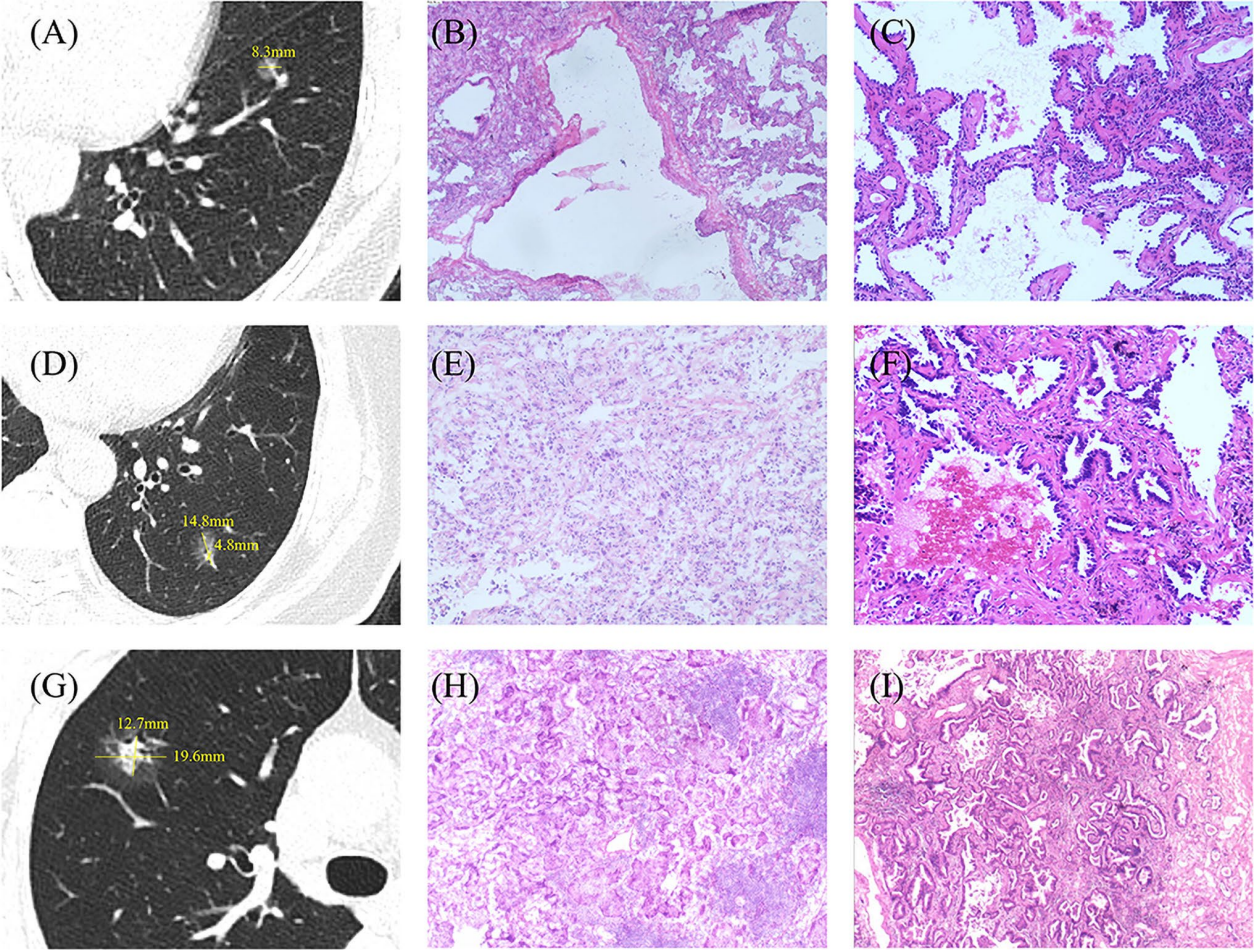
CT and pathological sections for the three groups. **A**–**C** are the chest CT, × 100 frozen section, and × 100 paraffin section for a patient with both FS and FP diagnosed as AIS. **D**–**F** represent the chest CT, × 100 frozen section, and × 100 paraffin section for a patient diagnosed with MIA in FS and IAC in FP. **G**–**I** depict the chest CT, × 100 frozen section, and × 100 paraffin section for a patient with FS and FP diagnosed as IAC

This study first looked for common features of FS misdiagnosis from CT images. We found that for patients diagnosed with AAH/AIS/MIA by FS, bronchial inflation sign, vascular convergence sign, lobulation sign, spiculation sign, and the diameter of the nodule are independent risk factors for pathological upgrading. When studying IAC patients, those with nodules ≤ 12.6 mm and CTR ≤ 0.5 are prone to be underestimated during FS diagnosis, perhaps due to their small solid components, which might be missed during pathological sectioning. Based on this, we constructed a model to predict pathological upgrading. In this study, this model identified 72.6% of patients upgraded from FS to IAC. For patients at higher risk of upgrading, pathologists need to be more cautious when diagnosing non-IAC.When necessary, re-sectioning or pathological consultation may improve FS accuracy to some extent. (Fig. 4).

Existing studies have substantiated that pathologic prediction models established through CT imaging tend to have a higher concordance rate with FP than with FS [27]. However, FS possesses advantages over CT-based pathological predictions, such as offering explicit pathological evidence, verifying surgical margins, detecting pleural invasion, and identifying spread through air spaces [28]. We believe that FS can achieve higher accuracy by enhancing the FS slide preparation and reading techniques, like elastic staining and inflation techniques [29, 30]. Some studies have combined CT imaging features with FS results to build prediction models, showing greater accuracy than models solely based on CT imaging [31–33]. Improving the accuracy of FS would equally benefit the predictions of these models.

Our study has certain limitations. As a single-center retrospective investigation, conditions in other centers might differ, and the prospective validation of the conclusions remains to be conducted.

## Conclusion

CT imaging has the capacity to effectively detect patients at risk of upstaging during FS analysis, with substantial potential to enhance the precision of FS.

## Data Availability

The data supporting this study’s findings are available from the corresponding author upon reasonable request.

## References

[1] Siegel RL, Miller KD, Fuchs HE, Jemal A (2022). Cancer statistics, 2022. CA Cancer J Clin.

[2] Jonas DE, Reuland DS, Reddy SM, Nagle M, Clark SD, Weber RP, Enyioha C, Malo TL, Brenner AT, Armstrong C, Coker-Schwimmer M, Middleton JC, Voisin C, Harris RP (2021). Screening for lung cancer with low-dose computed tomography: updated evidence report and systematic review for the US Preventive Services Task Force. JAMA.

[3] Ito H, Suzuki K, Mizutani T, Aokage K, Wakabayashi M, Fukuda H, Watanabe SI, Japan Clinical Oncology Group Lung Cancer Surgical Study G (2020). Long-term survival outcome after lobectomy in patients with clinical T1 N0 lung cancer. J Thorac Cardiovasc Surg.

[4] Saji H, Okada M, Tsuboi M, Nakajima R, Suzuki K, Aokage K, Aoki T, Okami J, Yoshino I, Ito H, Okumura N, Yamaguchi M, Ikeda N, Wakabayashi M, Nakamura K, Fukuda H, Nakamura S, Mitsudomi T, Watanabe SI, Asamura H, West Japan Oncology G and Japan Clinical Oncology G (2022). Segmentectomy versus lobectomy in small-sized peripheral non-small-cell lung cancer (JCOG0802/WJOG4607L): a multicentre, open-label, phase 3, randomised, controlled, non-inferiority trial. Lancet.

[5] Suzuki K, Watanabe SI, Wakabayashi M, Saji H, Aokage K, Moriya Y, Yoshino I, Tsuboi M, Nakamura S, Nakamura K, Mitsudomi T, Asamura H, West Japan Oncology G and Japan Clinical Oncology G (2022). A single-arm study of sublobar resection for ground-glass opacity dominant peripheral lung cancer. J Thorac Cardiovasc Surg.

[6] Russell PA, Wainer Z, Wright GM, Daniels M, Conron M, Williams RA (2011). Does lung adenocarcinoma subtype predict patient survival? A clinicopathologic study based on the new International Association for the Study of Lung Cancer/American Thoracic Society/European Respiratory Society international multidisciplinary lung adenocarcinoma classification. J Thorac Oncol.

[7] Travis WD, Brambilla E, Noguchi M, Nicholson AG, Geisinger KR, Yatabe Y, Beer DG, Powell CA, Riely GJ, Van Schil PE, Garg K, Austin JH, Asamura H, Rusch VW, Hirsch FR, Scagliotti G, Mitsudomi T, Huber RM, Ishikawa Y, Jett J, Sanchez-Cespedes M, Sculier JP, Takahashi T, Tsuboi M, Vansteenkiste J, Wistuba I, Yang PC, Aberle D, Brambilla C, Flieder D, Franklin W, Gazdar A, Gould M, Hasleton P, Henderson D, Johnson B, Johnson D, Kerr K, Kuriyama K, Lee JS, Miller VA, Petersen I, Roggli V, Rosell R, Saijo N, Thunnissen E, Tsao M, Yankelewitz D (2011). International Association for the Study of Lung Cancer/American Thoracic Society/European Respiratory Society International multidisciplinary classification of lung adenocarcinoma. J Thorac Oncol.

[8] Fu F, Chen Z, Chen H (2023). Treating lung cancer: defining surgical curative time window. Cell Res.

[9] Ishida H, Shimizu Y, Sakaguchi H, Nitanda H, Kaneko K, Yamazaki N, Yanagihara A, Taguchi R, Sakai F, Yasuda M, Kobayashi K (2019). Distinctive clinicopathological features of adenocarcinoma in situ and minimally invasive adenocarcinoma of the lung: a retrospective study. Lung Cancer.

[10] Cao J, Yuan P, Wang Y, Xu J, Yuan X, Wang Z, Lv W, Hu J (2018). Survival rates after lobectomy, segmentectomy, and wedge resection for non-small cell lung cancer. Ann Thorac Surg.

[11] Dolan D, Swanson SJ, Gill R, Lee DN, Mazzola E, Kucukak S, Polhemus E, Bueno R, White A (2022). Survival and recurrence following wedge resection versus lobectomy for early-stage non-small cell lung cancer. Semin Thorac Cardiovasc Surg.

[12] Liu S, Wang R, Zhang Y, Li Y, Cheng C, Pan Y, Xiang J, Zhang Y, Chen H, Sun Y (2016). Precise diagnosis of intraoperative frozen section is an effective method to guide resection strategy for peripheral small-sized lung adenocarcinoma. J Clin Oncol.

[13] Zhang Y, Fu F, Chen H (2020). Management of ground-glass opacities in the lung cancer spectrum. Ann Thorac Surg.

[14] Li F, Yang L, Zhao Y, Yuan L, Wang S, Mao Y (2019). Intraoperative frozen section for identifying the invasion status of lung adenocarcinoma: a systematic review and meta-analysis. Int J Surg.

[15] Shima T, Kinoshita T, Sasaki N, Uematsu M, Sugita Y, Shimizu R, Harada M, Hishima T, Yamamoto A, Horio H (2021). Feasibility of intraoperative diagnosis of lung adenocarcinoma in situ to avoid excessive resection. J Thorac Dis.

[16] Aokage K, Suzuki K, Saji H, Wakabayashi M, Kataoka T, Sekino Y, Fukuda H, Endo M, Hattori A, Mimae T, Miyoshi T, Isaka M, Yoshioka H, Nakajima R, Nakagawa K, Okami J, Ito H, Kuroda H, Tsuboi M, Okumura N, Takahama M, Ohde Y, Aoki T, Tsutani Y, Okada M, Watanabe SI, Japan Clinical Oncology G (2023). Segmentectomy for ground-glass-dominant lung cancer with a tumour diameter of 3 cm or less including ground-glass opacity (JCOG1211): a multicentre, single-arm, confirmatory, phase 3 trial. Lancet Respir Med.

[17] Zhu E, Xie H, Dai C, Zhang L, Huang Y, Dong Z, Guo J, Su H, Ren Y, Shi P, Fu R, Qin S, Wu C, Chen C (2018). Intraoperatively measured tumor size and frozen section results should be considered jointly to predict the final pathology for lung adenocarcinoma. Mod Pathol.

[18] He P, Yao G, Guan Y, Lin Y, He J (2016). Diagnosis of lung adenocarcinoma in situ and minimally invasive adenocarcinoma from intraoperative frozen sections: an analysis of 136 cases. J Clin Pathol.

[19] Wu G, Woodruff HC, Sanduleanu S, Refaee T, Jochems A, Leijenaar R, Gietema H, Shen J, Wang R, Xiong J, Bian J, Wu J, Lambin P (2020). Preoperative CT-based radiomics combined with intraoperative frozen section is predictive of invasive adenocarcinoma in pulmonary nodules: a multicenter study. Eur Radiol.

[20] Jiang Y, Che S, Ma S, Liu X, Guo Y, Liu A, Li G, Li Z (2021). Radiomic signature based on CT imaging to distinguish invasive adenocarcinoma from minimally invasive adenocarcinoma in pure ground-glass nodules with pleural contact. Cancer Imaging.

[21] Huang L, Lin W, Xie D, Yu Y, Cao H, Liao G, Wu S, Yao L, Wang Z, Wang M, Wang S, Wang G, Zhang D, Yao S, He Z, Cho WC, Chen D, Zhang Z, Li W, Qiao G, Chan LW, Zhou H (2022). Development and validation of a preoperative CT-based radiomic nomogram to predict pathology invasiveness in patients with a solitary pulmonary nodule: a machine learning approach, multicenter, diagnostic study. Eur Radiol.

[22] Naidich DP, Bankier AA, MacMahon H, Schaefer-Prokop CM, Pistolesi M, Goo JM, Macchiarini P, Crapo JD, Herold CJ, Austin JH, Travis WD (2013). Recommendations for the management of subsolid pulmonary nodules detected at CT: a statement from the Fleischner Society. Radiology.

[23] Zhang Y, Deng C, Fu F, Ma Z, Wen Z, Ma X, Wang S, Li Y, Chen H (2021). Excellent prognosis of patients with invasive lung adenocarcinomas during surgery misdiagnosed as atypical adenomatous hyperplasia, adenocarcinoma in situ, or minimally invasive adenocarcinoma by frozen section. Chest.

[24] McCarthy DP, DeCamp MM (2021). Does the punishment fit the crime? Using frozen section results to guide extent of resection. Chest.

[25] Su H, Gu C, She Y, Xu L, Yang P, Xie H, Zhao S, Wu C, Xie D, Chen C (2021). Predictors of upstage and treatment strategies for stage IA lung cancers after sublobar resection for adenocarcinoma in situ and minimally invasive adenocarcinoma. Transl Lung Cancer Res.

[26] Yeh YC, Nitadori J, Kadota K, Yoshizawa A, Rekhtman N, Moreira AL, Sima CS, Rusch VW, Adusumilli PS, Travis WD (2015). Using frozen section to identify histological patterns in stage I lung adenocarcinoma of </= 3 cm: accuracy and interobserver agreement. Histopathology.

[27] Lv YL, Zhang J, Xu K, Jin XY, Zhang XB, Yang HH, Fan XH, Zhang YJ, Li M, Zheng ZC, Huang J, Ye XD, Tao GY, Han YC, Ye B (2022). Computed tomography versus frozen sections for distinguishing lung adenocarcinoma: A cohort study of concordance rate. Asian J Surg.

[28] Owen RM, Force SD, Gal AA, Feingold PL, Pickens A, Miller DL, Fernandez FG (2013). Routine intraoperative frozen section analysis of bronchial margins is of limited utility in lung cancer resection. Ann Thorac Surg.

[29] Myung JK, Choe G, Chung DH, Seo JW, Jheon S, Lee CT, Chung JH (2008). A simple inflation method for frozen section diagnosis of minute precancerous lesions of the lung. Lung Cancer.

[30] Xiang Z, Zhang J, Zhao J, Shao J, Zhao L, Zhang Y, Qin G, Xing J, Han Y, Yu K (2020). An effective inflation treatment for frozen section diagnosis of small-sized lesions of the lung. J Thorac Dis.

[31] Xinli W, Xiaoshuang S, Chengxin Y, Qiang Z (2022). CT-assisted improvements in the accuracy of the intraoperative frozen section examination of ground-glass density nodules. Comput Math Methods Med.

[32] Wang B, Tang Y, Chen Y, Hamal P, Zhu Y, Wang T, Sun Y, Lu Y, Bhuva MS, Meng X, Yang Y, Ai Z, Wu C, Sun X (2020). Joint use of the radiomics method and frozen sections should be considered in the prediction of the final classification of peripheral lung adenocarcinoma manifesting as ground-glass nodules. Lung Cancer.

[33] Sun Y, Wang B, Bi K, Meng X, Zhang L, Sun X (2020). The combined nomogram based on the CT features may be used as a complementary method of frozen sections to predict invasive lung adenocarcinoma manifesting as ground-glass nodules. J Thorac Dis.

